# Senescence State in Mesenchymal Stem Cells at Low Passages: Implications in Clinical Use

**DOI:** 10.3389/fcell.2022.858996

**Published:** 2022-04-04

**Authors:** Raquel M. Alves-Paiva, Sabrina do Nascimento, Denise De Oliveira, Larissa Coa, Kelen Alvarez, Nelson Hamerschlak, Oswaldo Keith Okamoto, Luciana C. Marti, Andrea T. Kondo, Jose Mauro Kutner, Maria Augusta Tezelli Bortolini, Rodrigo Castro, Juliana A. Preto de Godoy

**Affiliations:** ^1^ Department of Hemotherapy and Cellular Therapy, Hospital Israelita Albert Einstein, São Paulo, Brazil; ^2^ Experimental Research Laboratory, Hospital Israelita Albert Einstein, São Paulo, Brazil; ^3^ Human Genome and Stem Cell Research Center, Department of Genetics and Evolutionary Biology, Biosciences Institute, University of São Paulo (USP), Sao Paulo, Brazil; ^4^ Paulista School of Medicine, Federal University of São Paulo, São Paulo, Brazil

**Keywords:** mesenchymal stem cells, senescence, telomere, differentiation, clinical use

## Abstract

Mesenchymal stem cells (MSCs) are multipotent cells found in various tissues and are easily cultivated. For use in clinical protocols, MSCs must be expanded to obtain an adequate number of cells, but a senescence state may be instituted after some passages, reducing their replicative potential. In this study, we report a case where MSC derived from an elderly donor acquired a senescence state after three passages. The bone marrow was aspirated from a female patient submitted to a cell therapy for the incontinency urinary protocol; MSCs were cultivated with DMEM low glucose, supplemented with 10% autologous serum (AS) plus 1% L-glutamine and 1% antibiotic/antimycotic. Senescence analysis was performed by β-galactosidase staining after 24 and 48 h. Controls were established using BM-MSC from healthy donors and used for senescence and gene expression assays. Gene expression was performed using RT-PCR for pluripotency genes, such as *SOX2*, *POU5F1*, *NANOG*, and *KLF4.* MSC telomere length was measured by the Southern blotting technique, and MSCs were also analyzed for their capacity to differentiate into adipocytes, chondrocytes, and osteocytes. The patient’s MSC expansion using AS displayed an early senescence state. In order to understand the role of AS in senescence, MSCs were then submitted to two different culture conditions: 1) with AS or 2) with FBS supplementation. Senescence state was assessed after 24 h, and no statistical differences were observed between the two conditions. However, patients’ cells cultured with AS displayed a higher number of senescence cells than FBS medium after 48 h (*p* = 0.0018). Gene expression was performed in both conditions; increased expression of *KLF4* was observed in the patient’s cells in comparison to healthy controls (*p* = 0.0016); reduced gene expression was observed for *NANOG* (*p* = 0.0016) and *SOX2* (*p* = 0.0014) genes. Telomere length of the patient’s cells was shorter than that of a healthy donor and that of a patient of similar age. Osteocyte differentiation seemed to be more diffuse than that of the healthy donor and that of the patient of similar age. MSCs could enter a senescence state during expansion in early passages and can impact MSC quality for clinical applications, reducing their efficacy when administered.

## 1 Introduction

Mesenchymal stem cells (MSCs) are non-hematopoietic cells capable of self-renewal and differentiation into cells of mesodermal origin. They are easily isolated from several tissues/organs and can be largely expanded under culture conditions. According to the International Society for Cell and Gene Therapy (ISCT), three minimal criteria were proposed for defining MSCs: 1) adherence to plastic; 2) surface antigen expression where MSC must express CD90, CD73, and CD105 and no expression for CD45, CD34, CD14 or CD11b, CD19 or CD79α, and HLA-DR; 3) multipotent differentiation potential where MSCs should be able to differentiate *in vitro* into osteoblasts, adipocytes, and chondroblasts (Dominici et al., 2006).

MSCs are being widely used as a source of adult stem cells for therapy of several diseases such as graft versus host disease (Le Blanc et al., 2004), Crohn’s disease (García-Olmo et al., 2005), multiple sclerosis (Connick et al., 2012), and COVID-19 (Shi et al., 2021). Most clinical protocols use high cell doses per patient which has an impact on MSC culture due to the requirement of a high replicative rate and numerous cell passages to achieve the desired dose.

MSCs have a limited lifespan *in vitro* and, after some cell divisions, they enter a state of senescence that is characterized by irregular cell morphology and a decrease in proliferation rate (Wagner et al., 2008). However, a senescence state could also be achieved in initial passages; senescence in dividing cells after a period of normal growth is followed by a proliferation cease. This phenomenon is accompanied by significant cellular changes such as the typical Hayflick phenomenon, a decrease in proliferation, telomere shortening, and impairment of functional properties (Bonab et al., 2006). The typical Hayflick phenomenon is a concept that helps explain some events that lead to aging of normal cells (Shay and Wright, 2000).

This study discusses a case where bone marrow-derived MSCs from a patient enrolled in a clinical protocol decreased their proliferation rate in an initial passage and the impact on the quality and number of cells intended to be delivered to the patient.

## 2 Materials and Methods

### 2.1 Patients

MSCs were isolated from the bone marrow from the iliac crest of three patients (56, 52, and 60 years old) initially enrolled in a cell therapy protocol approved by the National Ethics Research Commission (Comissão Nacional de Ética em Pesquisa/CAAE: 18150613.7.3001.0071). MSCs from a healthy donor (34 years old) were used in differentiation, senescence assay, and gene expression; the telomere length was performed using cells from a patient that presented senescence, from the healthy donor, and from two other participants in the cell therapy protocol (52 and 60 years old). No data from this cell therapy trial were presented in this article; we presented a case report related to a senescence state that could impact the clinical use of these cells. Cells from four different patients were used for performing assays and are summarized in Table 1, and the experiments will be identified according to this table from now on.

**TABLE 1:** Patients’ cells used in different assays in the study.

Patient	Age (years)	Assays	Observations
#1	56	Differentiation, doubling time, cytometry, Southern blotting, beta-galactosidase, and gene expression	Senescent
#2	60	Differentiation, doubling time, cytometry, and Southern blotting	No senescent
#3	34	Differentiation, doubling time, cytometry, Southern blotting, beta-galactosidase, and gene expression	No senescent
#4	52	Southern blotting	No senescent

### 2.2 Mesenchymal Stem Cell Culture

Bone marrow mononuclear cells were separated by Ficoll-Paque Plus gradient density (GE Healthcare) and MSCs were cultivated in DMEM low glucose (DMEM-LG) supplemented with 1% antibiotic–antimycotic solution (10,000 units/mL of penicillin, 10,000 μg/ml of streptomycin, and 25 μg/ml of amphotericin B), 1% L-glutamine 200 mM, and 10% fetal bovine serum (FBS) or 10% autologous serum (AS) in T75 flasks at 37°C in a humidified 5% CO_2_ atmosphere. Non-adherent cells were discarded after 48 h; the adherent layer was washed twice with DMEM-LG and maintained in culture until the 3^rd^ passage. The medium was changed every other day, and cell harvesting was performed using TrypLE™ Express (Gibco) for 5 min at 37°C and inactivated by complete medium; centrifugation was performed, and cells were counted. Cell viability was assessed by trypan blue staining (Sigma-Aldrich, St. Louis, MO, United States).

### 2.3 Immunophenotyping

As the release criteria, MSCs must exhibit specific cell surface expression profile: positive for CD105 (FITC or PE mouse anti-human CD105—endoglin, BD Pharmingen, clone 266), CD73 (PE mouse anti-human CD73, BD Pharmingen, clone AD2), and CD90 (PE mouse anti-human CD90, BD Pharmingen, clone 5E10), and negative for hematopoietic markers CD14 (APC mouse anti-human CD14, BD Pharmingen, clone M5E2), CD34 (PE mouse anti-human CD34, BD Pharmingen, clone 4H11), CD45 (FITC mouse anti-human CD45, BD Pharmingen, clone HI30), CD19 (PE-Cy7 mouse anti-human CD19, BD Pharmingen, SJ25C1), and HLA-DR (PerCP-Cy5.5 mouse anti-human HLA-DR, BD Pharmingen, clone LN3) surface molecule (Dominici et al., 2006). Cells in passage 3 were resuspended in a staining solution (PBS supplemented with 1% FBS and 0.05% azide). Staining was performed for 30 min at room temperature in the dark. The data were acquired using a FACS Canto II flow cytometer (BD Biosciences, San Jose, CA) and analyzed by Kaluza software (Beckman-Coulter). At least 10,000 events were acquired for each sample.

### 2.4 Cell Lineage Differentiation

After the establishment of MSC cultures on the 3^rd^ passage, the cells were differentiated into adipocytes, osteoblasts, and chondrocytes.

#### 2.4.1 Adipocyte Differentiation

MSCs were differentiated into adipocyte lineage, for that cells at P3 were plated in 12-well culture plates (Corning, St. Louis, MO, United States) in triplicates, one as a negative control, at a density of 4 × 104 cells/well for 48 h before changing to a specific medium for adipogenic induction (StemPro^®^ Adipogenesis Differentiation Kit). The medium was changed every three days, and the negative control was kept in complete DMEM-LG. After 21 days, the cells were fixed in 4% paraformaldehyde, washed with PBS, and stained. Intracellular lipid granules were visualized after staining with 0.3% Oil Red O stain (Fisher Scientific, New Hampshire, United States). The plates were analyzed using an inverted microscope (EVOS M5000, Invitrogen).

#### 2.4.2 Osteocyte Differentiation

MSCs were differentiated into osteogenic lineages, for which cells (P3) were seeded in triplicate, one as a negative control, onto 12-well plates at a density of 2 × 104 cells/well. After 48 h, the medium was switched to an inducing medium (StemPro Osteogenesis Differentiation Kit) or maintained in regular growth medium for a negative control sample. After 21 days, cells were fixed in 4% paraformaldehyde and Ca^2+^ deposits stained with 1% Alizarin Red S (Acros Organics, New Jersey, United States). The plates were analyzed using an inverted microscope (EVOS M5000, Invitrogen).

#### 2.4.3 Chondrocyte Differentiation

MSC cells were differentiated into chondrogenic lineage; for that, cells (P3) were seeded in triplicate, one well as a negative control, onto 12-well plates at a cell density of 3,75 × 106 cells/mL to generate a micromass seeding of 5-μL droplets of cell solution in the center of each well. After 2 h, the medium was switched to an inducing medium (StemPro Condrogenesis Differentiation Kit) or maintained in regular growth medium for a negative control sample. After 21 days, cells were fixed in 4% paraformaldehyde and proteoglycan deposits stained with Alcian blue stain (Fisher Scientific, New Hampshire, United States). The plates were analyzed using an inverted microscope (EVOS M5000, Invitrogen).

### 2.5 Cumulative Population Doublings

Population doublings were calculated for each MSC culture (#1, #2, and #3) using the following equation:

$$\log10\;\left(\frac{N}{No}\right)x\;3.33,$$

where N is the number of cells at harvest and N_0_ is the number of cells plated. Cumulative population doublings were calculated for each passage as the sum of the current and all the previous population doubling values.

### 2.6 Cellular Senescence Assay

MSCs from patients 1 and 3 were seeded at a density of 2 × 104 cells/well and cultured in complete DMEM-LG for 24 and 48 h; some wells were cultivated with 10% FBS and others with 10% autologous serum. The activity associated with β-galactosidase was analyzed by the Senescence β-Galactosidase Staining Kit (Cell Signaling, Danver, MA, United States) following the manual instructions.

### 2.7 Gene Expression by qRT-PCR

#### 2.7.1 Total RNA Extraction and cDNA Synthesis

Total RNA was isolated using the RNeasy^®^ Mini kit (Qiagen) following the manufacturers’ instructions. The purified total RNA quality was assessed by spectrophotometry using Nanodrop (Thermo Scientific). A total volume of 1 μg of the extracted RNA was reverse transcribed into cDNA using the Quantitec Reverse Transcription Kit (Qiagen).

#### 2.7.2 Quantitative Real-Time Polymerase Chain Reaction

PCR amplifications were performed on the ABI Prism 7500 (Applied Biosystems). The amplification program consisted of initial denaturation at 95°C for 1 min, followed by 40 cycles at 95°C for 10 s, and an annealing and extension phase at 60°C for 30 s. The qRT-PCR assays were performed in technical triplicates, the relative expression levels of each gene were normalized to GAPDH using the 2-ΔΔ Ct method. The gene-specific primers used for amplification using the QuantiFast^®^ SYBR^®^ Green PCR Kit and their sequences are described in Table 2.

**TABLE 2:** Sequence of gene-specific primers used for qRT-PCR.

Primer	Sequence	Concentration (nM)
***GAPDH***	Forward: GAGAAGGCTGGGGCTCA	400
	Reverse: GTC​CTT​CCA​CGA​TAC​CAA​A	
***SOX2***	Forward: TGG​GAG​GGG​TGC​AAA​AGA​GG	250
	Reverse: GAG​TGT​GGA​TGG​GAT​TGG​TG	
***POU5F1***	Forward: AGA​AGT​GGG​TGG​AGG​AAG​CTG​ACA​A	800
	Reverse: TGG​GTT​TCG​GGC​ACT​GCA​GG	
***NANOG***	Forward: CCT​ATG​CCT​GTG​ATT​TGT​GG	250
	Reverse: CTG​GGA​CCT​TGT​CTT​CCT​TT	
***KLF4***	Forward: ACC​TAC​ACA​AAG​AGT​TCC​CAT​C	400
	Reverse: ATCTGAGCGGGCGAATTT	

### 2.8 Telomere Length

DNA was extracted using the DNeasy Blood and Tissue Kit (Qiagen, Germantown, MD, United States). Mean telomere length was measured using MSC DNA by Southern blotting [TeloTAGGG Telomere Length Assay (Roche)], as previously described by Gutierrez-Rodrigues (2014). Briefly, 800 ng genomic DNA was digested by FastDigest HinfI and RsaI (Thermo Scientific, Waltham, MA, United States) at 37°C for 2 h. Following digestion, DNA fragments were electrophoresed for 5 h on a 0.8% agarose gel, denatured, neutralized, and transferred to a nylon membrane for Southern blot analysis with proprietary digoxigenin (DIG)-labeled probes and chemiluminescent substrates. We calculated the mean TRF length using the equation Ʃ(ODi)/Ʃ(ODi/Li), where ODi represents the chemiluminescent signal and Li is the fragment length at a given position. For the experiment, a reference sample was included. This assay was performed by an external service (University of Sao Paulo—Ribeirao Preto).

### 2.9 Statistical Analysis

Data were presented as mean ± SEM. The Mann–Whitney non-parametric test or Student’s t test with or without Welch’s correction was used, when appropriate, for comparison between groups, using GraphPad Prism 5.00 (GraphPad Software). *p* ≤ 0.05 was considered statistically significant.

## 3 Results

The patient’s bone marrow-derived MSCs were grown as described in Section 2.2. The patient, female, 60 years old, was included in the clinical trial due to urinary stress incontinence. In the protocol, it was established that MSCs should be cultivated with AS up to passage five in order to achieve 10 million cells to be infused after releasing tests were performed according to local guidelines (Table 3). According to the National Health Surveillance Agency (ANVISA) guidelines (RDC 508/2021), some guidelines should be followed in order to release cells for clinical use: microbiological examinations, endotoxin assay, mycoplasma detection by RT-PCR, karyotyping, immunophenotyping, and potency assay (differentiation).

**TABLE 3:** Quality control of MSCs.

Assay	Expected	Patient
Endotoxin	<5 EU/mL	<5 EU/mL
*Mycoplasma*	Negative	Negative
Bacterial culture	Negative	Negative
Karyotyping	46, XX	46, XX
	46, XY	
MSC differentiation	Adipocyte, chondrocyte, and osteocyte	Adipocyte, chondrocyte, and osteocyte
Immunophenotyping	CD73, CD90, and CD105 (positive)	CD73, CD90, and CD105 (positive)
	CD11b, CD14, CD19, CD34, CD45, CD79a, and HLA-DR (negative)	CD11b, CD14, CD19, CD34, CD45, CD79a, and HLA-DR (negative)

**FIGURE 1:**
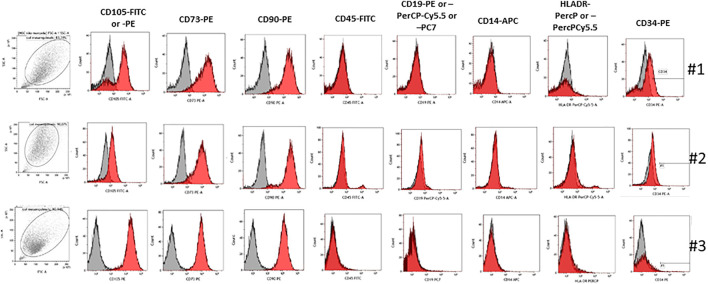
Immunophenotyping of MSCs from the healthy donor and senescence patient. Representative histograms of expression for MSCs derived from bone marrow from the healthy donor and senescence patient. Cells stained positive for CD90, CD105, and CD73, and they were negative for CD14, CD19, CD45, CD34, and HLA-DR.

Cells from patients 1, 2, and 3 stained positively for canonical MSC surface markers CD90, CD105, and CD73, whereas they were negative for CD45, CD19, CD14, and HLA-DR (Figure 1). However, #1 cells presented a decrease in CD105 expression where there was a negative population for this marker; 71.34% of CD105 expression for #1 patient when compared to #3 patient who presented 99.35%. Patient #2 also presented a decrease in CD105 expression (80.83 *vs.* 99.35%) (Table 4). The expression of CD34 was increased in #1 cells (15.84%) in relation to #2 (0.61%) and #3 (1.05%) (Table 4).

**TABLE 4:** Marker expression profile of MSCs.

Marker	#1	#2	#3
CD105	71,34	80,83	99,35
CD73	93,23	94,31	99,56
CD90	96,9	95,87	99,62
CD45	0,18	4,61	0,44
CD19	0,36	1,02	1,16
CD14	0,18	3	0,43
HLA-DR	5,14	4,44	1,39
CD34	15,84	0,61	1,05

**FIGURE 2:**
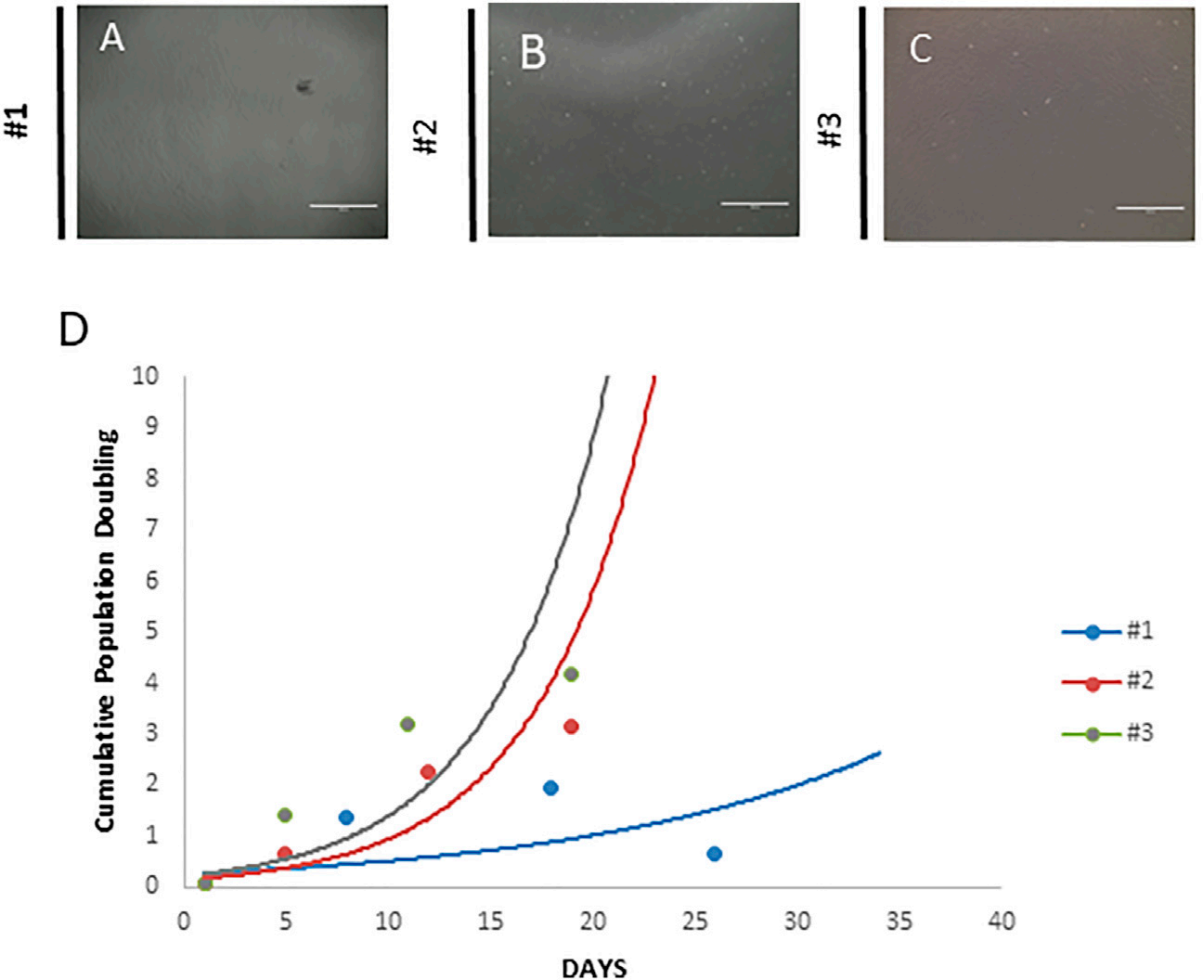
Morphology of the mesenchymal stem cell cultures. **(A)** Mesenchymal stem cells from the patient (senescence). The cells show fibroblast morphology, but in higher passages, this pattern seems to be less evident, suggesting a senescence state. **(B)** Mesenchymal stem cells from the healthy donor. These cells exhibited a typical fibroblastoid morphology, and it is not lost even in high passages. **(C)** Mesenchymal stem cells from the patient (no senescence). These cells exhibited a typical fibroblastoid morphology, and it is not lost even in high passages. **(D)** Cumulative cell population doubling. Both cultures were analyzed using the following formula: log_10_ (N/N_0_) x 3.33, where N is the number of cells at the harvesting and N_0_ is the number of cells plated. Scale bar = 1,000 µm.

In this case report, MSC culture supplemented with 10% AS presented a senescence state at passage 3. According to Hayflick and Moorhead (1961), cells enter a state called replicative senescence after some passages, being they stop dividing the main characteristic. As observed in our case, the cumulative population doubling level (CPDL) was calculated according to the following formula: log_10_ (N/N_0_) x 3.33, where N is the number of cells at the harvesting and N_0_ is the number of cells plated (Table 5). MSCs from the patients #1, #2, and #3 were cultivated according to a standard operation procedure and CPDL calculated. As observed in Figure 2, #1 (Figure 2A), #2 (Figure 2B), and #3 (Figure 2C), patients’ MSCs showed the same morphology; however, senescence changed the cell proliferation rate as seen on graphic (Figure 2D). The CPDL showed a logarithmic rate in #2 and #3 patient’s cells and at the same time, patient #1 showed a more linear rate. Table 5 shows the cell density plated in the different cell cultures and in the different passages.

**TABLE 5:** Cell density (x10^6^) in different passages.

Patient	Passage 1	Passage 2	Passage 3	Passage 4	Passage 5	Observations
**#1**	2.95	7.68	11.52	1.87	31.68	26 days of culture
**#2**	5	0.77	1.1	12.75		34 days of culture
**#3**	14	37	128	257.6		19 days of culture

**FIGURE 3:**
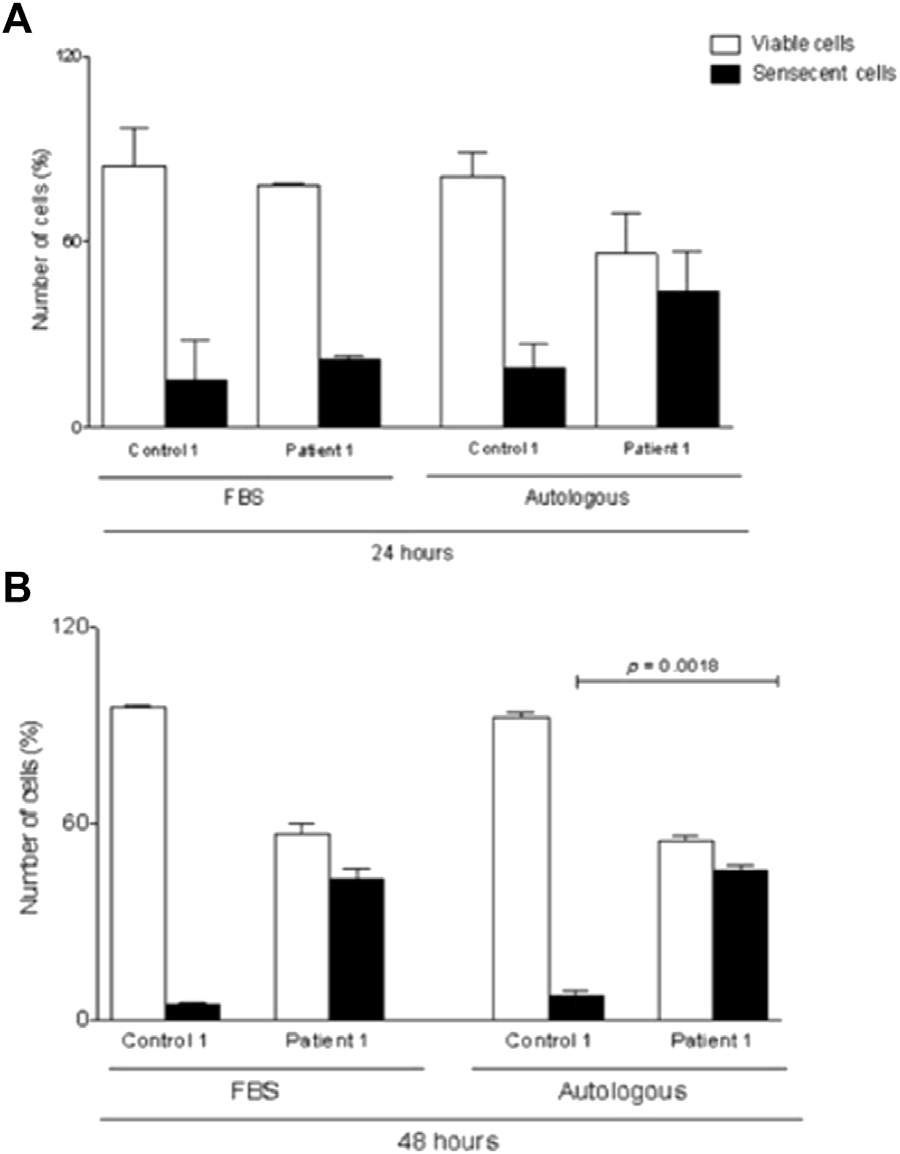
β-galactosidase staining of BM–MSCs from healthy control and patient maintained in culture for 24 and 48 h. **(A)** Staining of senescent cells from hMSC culture supplemented with 10% FBS and/or 10% autologous serum. **(B)** Quantification of senescent cells from hMSC culture supplemented with 10% FBS and/or 10% autologous serum. Data are mean ± SD. Statistical significance is shown using Student’s *t*-test analysis (*n* = 3); *p = 0.0019.

To find out the explanation about cell culture low rate growth, MSCs from patient #1 were also cultivated with 10% fetal bovine serum (FBS) used as standard. Additionally, MSCs from #3 patient were also cultured with 10% patient’s AS and 10% FBS up to passage 3. MSCs were then submitted to senescence assay, using a senescence β-galactosidase staining kit, and data were analyzed after 24 and 48 h. Figure 3 represents the senescent cells from both patients under 10% FBS and/or 10% AS conditions. The upper panel shows the MSC from #3 patient with a reduced number of senescent cells in comparison to the lower panel (#1 MSCs). The senescent profile was more pronounced under AS supplementation after 48 h in #1 patient’s cells in comparison to #3 MSCs (*p* = 0.0019) (Figure 3). However, #1 patient’s MSCs cultivated with FBS also presented senescence cells, suggesting that this event is more related to a cell factor instead to some soluble molecule.

**FIGURE 4:**
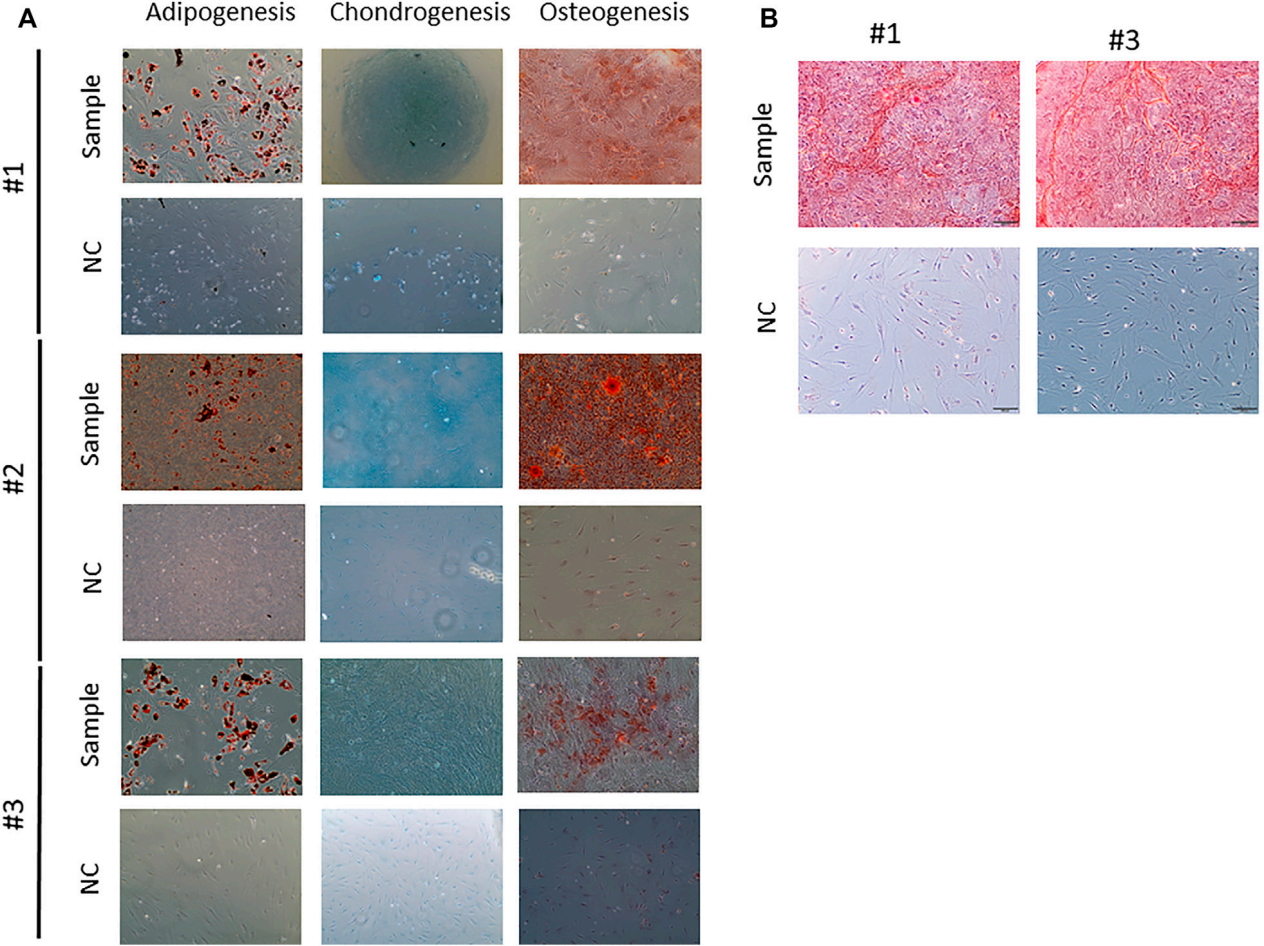
Multilineage Mesenchymal stem cell differentiation. **(A)** Adipogenesis differentiation. In negative control group (without adipogenic differentiation), no lipid droplets were observed. Lipid droplets are given in red. Chondrogenesis differentiation. In the control, no blue reaction was observed there is a slightly positivity showing the presence of proteoglicans. Osteogenesis differentiation. In the negative groups, no calcium deposits were observed. The healthy donor cells showed a strong presence of calcium deposits, and the patient cells exhibited a weak staining. 20-fold magnification. **(B)** Images of osteogenic differentiation from patients #1 and #3.

Surprisingly, #1 MSC differentiation into adipocytes and chondrocytes seemed to be normal (Figure 4A), but when analyzing the osteocyte differentiation, the staining seemed to be more diffuse when than that of #2 or #3 patient (Figure 4A). In the osteogenic differentiation, there is a non-specific staining and background in #1 patient; this may indicate that the cells are still at the beginning of osteogenic differentiation or the differentiation capacity is decreased. A better resolution image was obtained for the osteogenic differentiation from patients #1 and #3; as observed in Figure 4B, the osteogenic differentiation and staining with Alizarin red S showed more localized calcium deposits in patient #3 and also formed structures similar to lines; #1 cells showed calcium deposits more disorganized, without defined structures.

**FIGURE 5:**
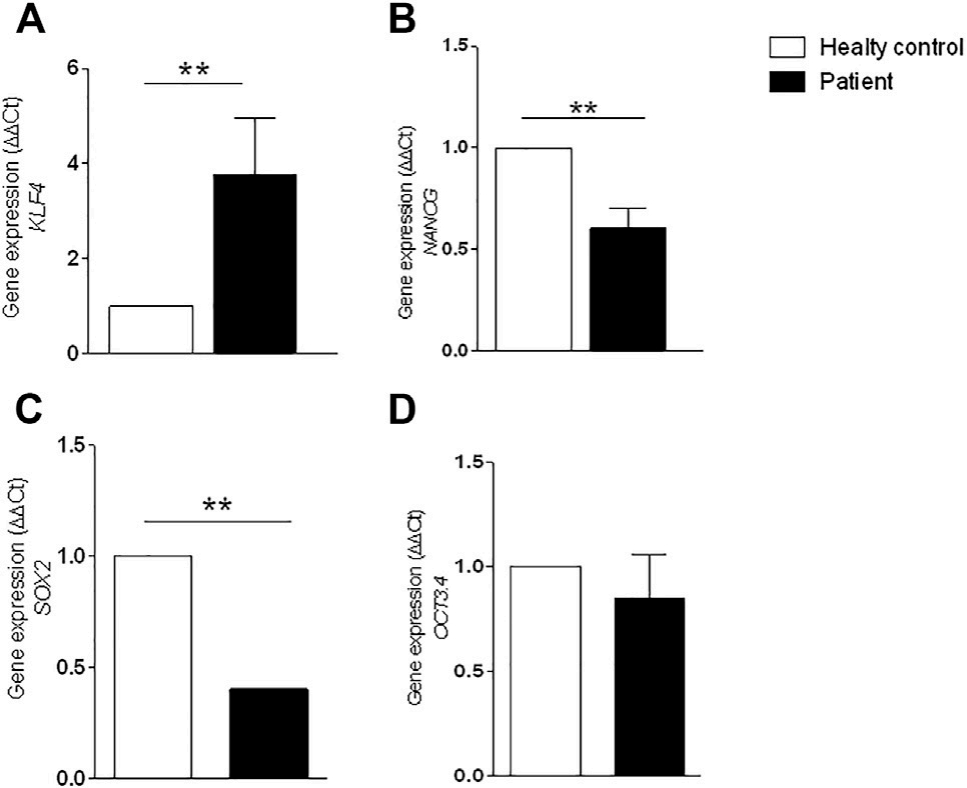
Gene expression by RT-PCR. Expression was analyzed using GAPDH reference gene and results were normalized through the control samples. **(A)** Increased expression of *KLF4* gene (** *p* = 0.0016); **(B)** reduced expression of *NANOG* gene (** *p* = 0.0016); **(C)** reduced expression of *SOX2* gene (** *p* = 0.0014); **(D)** reduced expression of *POU5F1 (OCT3.4)* gene. Error bars represent SD. Statistical significance was determined by Student’s t test. Data represent triplicate of 2 independent experiments.

To quantitatively compare the cells from patients #1 and #3, qRT-PCR was used to detect the expression of genes related to pluripotency. The attenuated cell proliferation from patient #1 cells was further evidenced by increased expression of *KLF4* (*p* = 0.0016) and reduced expression of *SOX2* (*p* = 0.0014), *OCT3.4*, and *NANOG* (*p* = 0.0016) (Figure 5).

Southern blot was performed to measure telomere length in the patient’s MSCs #1, #3, #5, and #7. Patients #1, #2, and #4 were enrolled in the same clinical trial for urinary incontinence. In this assay, the patients were nominated as patient 1 (#1), patient 2 (#3), patient 3 (#4), and healthy donor.

**FIGURE 6:**
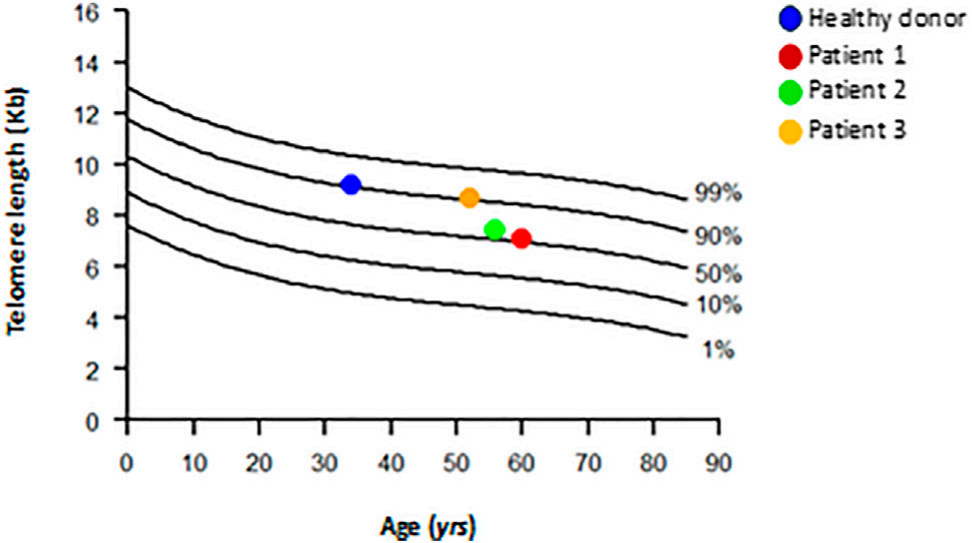
Distribution of age-matched telomere length in DNA from healthy subjects leukocytes and DNA from healthy subject and patients’ MSCs. Healthy subjects (*n* = 261) are represented as line; MSCs from patients and one healthy control are represented as colored circles. Telomere length is given as kilobases (kb) by Southern blot.

It is well known that MSCs have longer telomeres than leukocytes (Baxter et al., 2004; Fathi et al., 2019). Although we do not have a control curve for MSC, we purposely plotted the MSC data on the leukocyte curve of healthy donors (Figure 6). We found that the healthy MSC donor had a longer telomere (9.2 Kb; 90^th^ percentile) than the telomere of patients 1 and 2 (7.1 Kb and 7.4 Kb, respectively; 50^th^ percentile), and patient 3 presented a telomere length comparable to the healthy donor (8.7 Kb; 90^th^ percentile) (Table 6). It is worth noting that the patients were close in age and the control was younger. Therefore, we found that MSC from patients 1 and 2 have shorter telomeres than patient 3 and healthy donor.

**TABLE 6:** Telomere length by Southern blot analysis.

#ID	Age (years)	MSC telomere length (Kb)
Healthy donor	34	9.2
Patient 1^a^	60	7.1
Patient 2	56	7.4
Patient 3	52	8.7

a Patient related to this study (senescent cells).

## 4 Discussion

Senescence is a complex process that may require different approaches to alleviate or even prevent this profile and improve the clinical application of MSCs. In this study, we presented data from a single patient with uncommon MSC senescence at passage 3. Banfi et al. (2002) showed that bone marrow stromal cells display a decrease in telomere length when in culture, and despite their inability to maintain the telomere length (no telomerase activity was detected), these cells were able to undergo around 40 population doublings. Telomere shortening is observed over a lifetime in MSCs. The average telomere length in early-passage MSCs depends on the age of the donor, and it has been proposed that it ranges from 10 to 11 kb in cells from fetal to 7 kb in cells from postnatal (Guillot et al., 2007). Telomere-dependent MSC senescence has been confirmed; the growth stops when telomeres reach a length of 5.8–10.5 kb (Baxter et al., 2004). Although telomere shortening is a marker of cell senescence, as we have shown here, the length of telomeres varies from donor to donor, making telomere length an inconstant measure of MSC senescence (Li et al., 2017).

The differentiation capacity of stem cells is an important characteristic for the repair and regeneration of tissues/organs; however, the patient’s MSC differentiation into adipocytes and chondrocytes seemed to be normal, but the osteocyte differentiation may be reduced. Studies have been reported that the osteogenic activity of MSC is progressively reduced as a function of increasing lifespan (Banfi et al., 2000; Turinetto et al., 2016).

According to Martin et al. (1997), bone marrow stromal cells when cultivated using fibroblast growth factor-2 (FGF-2) were able to maintain their osteogenic potential after the *in vitro* expansion. However, Hagmann et al. (2013) showed that while maintaining its differentiation potential, the use of FGF-2 pre-selects some MSC phenotypes. Regarding the use of MSC in clinical protocols, the use of growth factors may not be the best option; some MSC subtypes can also account for its efficacy.

CD105 is involved in multiple functions of MSCs such as differentiation, angiogenesis, and regenerative potential (Aslan et al., 2006; Mark et al., 2013). Na et al. (2015) showed that the marker CD105 seems to be more sensitive than the others, CD73 and CD90, to viability-associated events, and CD105 may be used as an indicator of cell death. In addition, it can be an important marker to understand the mechanisms during MSC senescence. Corroborating our study, this marker was evaluated in the senescent MSC-derived microvesicles, and the results showed that senescent MSCs secreted microvesicles of smaller size, and the level of CD105^+^ also decreased (Lei et al., 2017). The senescence biomarker SA-β-gal is used to investigate the functional implications of aging in MSCs (Dimri et al., 1995). In this study, patients’ MSCs showed that a higher number of cells stained positive for SA-β-gal than healthy donors’ MSCs, especially after using AS as a supplement. Several studies have shown the importance of stem cells in repair and in regenerating tissue and organs. Differentiation capacity of young MSCs is superior to old cells, and old MSCs proliferated more slowly and became senescent due to telomere attrition (Kretlow et al., 2008; Li et al., 2017).

The molecule CD34 is a hematopoietic progenitor marker, and its expression is lost when the maturation process takes part (Hughes et al., 2020). The expression of CD34 is higher *in vivo* than in cultured MSCs, and some growth factors in culture medium could impact in its expression *in vitro* (Bellagamba et al., 2018; Viswanathan et al., 2019). An study showed a decrease in the proliferation rate in adventitial stromal cell-like cells, and it was related to the accumulation of CD105^+^/CD146^+^/CD271^+^ and also CD34^+^, which could represent a population that entered the cell cycle but did not complete the cell division (Braun et al., 2013). Patient #1 displayed a higher expression of CD34, which is not expected and could be related to an accumulation of non-proliferating cells, leading to the senescence state.

To evaluate the potential impairment of self-renewal and differentiation of MSC due to early senescence, we investigated the expression of genes related to pluripotency (*KLF4*, *OCT 3.4*, *NANOG*, and *SOX2*). An increased expression of *KLF4* was observed in the patient’s MSC culture in comparison to healthy controls.

The zinc finger protein Krüppel-like factor 4 (KLF4) is responsible for regulating gene transcription and cell fate, promoting malignant transformation, cell differentiation, tumor suppression, and stem cell properties (Kaczynski et al., 2003; Rowland and Peeper, 2006; Lin et al., 2011).

KLF4 is known to act in different ways by reducing proliferation rates by pathways such as p21^Waf1/Cip1^ and may be present in promoting malignant properties by suppressing p53 or by upregulation of NOTCH1 (Kaczynski et al., 2003; Lin et al., 2011). Direct blockage of KLF4 expression has been demonstrated to promote cell differentiation and reduce cell aging in MSCs (Rowland and Peeper, 2006). KLF4, a downstream mediator, can either repress or activate transcription and participate in cell cycle regulation and differentiation (Rowland et al., 2005; Li et al., 2013; Lu et al., 2019).

A decreased expression of *NANOG*, *SOX2*, and *OCT3.4* was also observed in the patient’s MSC in comparison to healthy controls. The reduced expression of these genes may indicate a reduced proliferation rate in the patient’s MSCs in comparison to healthy control MSCs.

NANOG is a transcription factor that is involved in the self-renewal of embryonic stem cells. The knockdown of *NANOG* and/or *OCT4* in MSCs has been shown to not maintain MSCs in an undifferentiated state. In contrast, the overexpression of OCT4 and NANOG in MSCs has been shown to increase the cell proliferation rate and differentiation potential and inhibit spontaneous differentiation, becoming essential markers for maintaining MSC properties (Shields et al., 1996).

POU5F1 (also known as OCT4 and OCT3.4) and SOX2 are essential transcription factors for pluripotency and self-renewal. Both genes are expressed in MSCs at low levels in early passages, and their levels gradually decrease as the passage number increases. The overexpression of OCT4/SOX2 in MSC has been shown to improve cell proliferation and differentiation. Many researchers have investigated the effects of these pluripotent genes on MSCs, but the results are still debatable. Forced expression of pluripotent cell-specific factors (Oct4, Sox2, Nanog, and cMyc) or combinations of these genes for reprogramming somatic or adult stem cells has been used to improve the pluripotency of MSCs (Zhang et al., 2000; Seo et al., 2009; Lin et al., 2011; Yoon et al., 2011; Han et al., 2014; Gawlik-Rzemieniewska et al., 2016). SOX2, implicated in the maintaining the stem cell potency of BM-MSCs (Otsubo et al., 2011), recently was reported in a senescence context; the co-culture of senescent endothelial cells with BM-MSCs was associated with an increased expression of miR-126a-3p, in association with a significant decrease of SOX2, targeted by miR-126a-3p (Lazzarini et al., 2019).

A previous study with BM-MSCs from different healthy young donors identified some markers that could predict the expansion capacity of MSCs before reaching senescence. In this study, for each early passage, the gene expression levels of pluripotency markers OCT4, NANOG, and SOX2 were correlated with the final population doubling (PD) number and, in agreement with our results, revealed that a high OCT4 gene expression might be a potential hallmark and predictor of MSCs’ lifespan *in vitro* (Piccinato et al., 2015). A recent study also showed that OCT4 maintains the self-renewal ability of MSCs and may reverse the senescence phenotype (Lu et al., 2019).

Low-passage cultures are recommended for clinical expansion of cultures (Lechanteur et al., 2016). Therefore, there is no explicit passage number provided for the 15 MSC products in clinical use, as resumed by Liu and contributors (2020). However, for MSCs to be clinically functional, it is essential to monitor the senescence of MSC aging. Recent studies have discussed and summarized some strategies for monitoring senescence, and the molecular mechanisms involved (Li et al., 2017; Liu and Chen, 2020). Although the topic has much clinical relevance, the current knowledge of senescence is mainly based on classical events, and sometimes, there are difficulties not previously described.

Further research is required to determine the approaches for safety and effectiveness in MSC-based cell therapy. MSC culture is quite simple; however, it is necessary to carefully monitor the expansion process to detect any potential changes that may interfere with clinical management. Improving the conditions for *ex vivo* expansion and monitoring of aging markers for MSCs would help suppress and monitor *in vitro* aging.

## Data Availability

The raw data supporting the conclusions of this article will be made available by the authors.
